# Linking political exposures to child and maternal health outcomes: a realist review

**DOI:** 10.1186/s12889-021-10176-2

**Published:** 2021-01-12

**Authors:** Maxwell S. Barnish, Si Ying Tan, Araz Taeihagh, Michelle Tørnes, Rebecca V. H. Nelson-Horne, G. J. Melendez-Torres

**Affiliations:** 1 Peninsula Technology Assessment Group (PenTAG), University of Exeter Medical School, Room 3.09f, South Cloisters, St Luke’s Campus, Heavitree Rd, Exeter, EX1 2LU UK; 2 Evidence Synthesis and Modelling for Health Improvement (ESMI), University of Exeter Medical School, Room 3.09f, South Cloisters, St Luke’s Campus, Heavitree Rd, Exeter, EX1 2LU UK; 3 Policy Systems Group (PSG), Lee Kuan Yew School of Public Policy, National University of Singapore, 16 Evans Road, Singapore, 259363 Singapore; 4 Ageing, Clinical and Experimental Research (ACER) Team, Institute of Applied Health Sciences, University of Aberdeen, Aberdeen, UK; 5 Independent Scholar, Glasgow, UK

**Keywords:** Child health, Maternal health, Health policy, International health, Politics, Realist synthesis

## Abstract

**Background:**

Conceptual and theoretical links between politics and public health are longstanding. Internationally comparative systematic review evidence has shown links between four key political exposures – the welfare state, political tradition, democracy and globalisation – on population health outcomes. However, the pathways through which these influences may operate have not been systematically appraised. Therefore, focusing on child and maternal health outcomes, we present a realist re-analysis of the dataset from a recent systematic review.

**Methods:**

The database from a recent systematic review on the political determinants of health was used as the data source for this realist review. Included studies from the systematic review were re-evaluated and those relating to child and/or maternal health outcomes were included in the realist synthesis. Initial programme theories were generated through realist engagement with the prior systematic review. These programme theories were adjudicated and refined through detailed engagement with the evidence base using a realist re-synthesis involving two independent reviewers. The revised theories that best corresponded to the evidence base formed the final programme theories.

**Results:**

Out of the 176 included studies from the systematic review, a total of 67 included child and/or maternal health outcomes and were included in the realist re-analysis. Sixty-three of these studies were ecological and data were collected between 1950 and 2014. Six initial programme theories were generated. Following theory adjudication, three theories in revised form were supported and formed the final programme theories. These related to a more generous welfare state leading to better child and maternal health especially in developed countries through progressive social welfare policies, left-of-centre political tradition leading to lower child mortality and low birth weight especially in developed countries through greater focus on welfare measures, and increased globalisation leading to greater child and infant mortality and youth smoking rates in LMECs through greater influence of multinational corporations and neoliberal trade organisations.

**Conclusion:**

We present a realist re-analysis of a large systematically identified body of evidence on how four key political exposures – the welfare state, democracy, political tradition and globalisation – relate to child and maternal health outcomes. Three final programme theories were supported.

**Supplementary Information:**

The online version contains supplementary material available at 10.1186/s12889-021-10176-2.

## Background

### Links between politics and population health

Politics has been described as ‘the practice of the art of science of directing and administrating states’ [1]. Conceptual and theoretical links between population health and politics are longstanding. One of the founding fathers of social medicine, the German physician, anthropologist and politician Rudolph Virchow (1812–1902) stated that ‘medicine is a social science, and politics nothing but medicine at a larger scale’ [2]. Moreover, Friedrich Engels, who played an important role in the development of Marxist political theory, published the socio-political treatise ‘A history of the working class in England’ [3], which has been described as the first, or among the first, works of modern public health research. Despite the existence of formal evidence-based systems for the licensing of medicines and medical devices especially in developed countries, many of the pathways through which influences on population health are generated and diffused are political [4, 5] and the influence of ideology can lead to marked evidence-policy gaps in health policy and other policy relevant to health [6]. Indeed, this may be seen as inevitable in a democracy where ‘politics has primacy’ and decisions can ‘never solely be made on evidence’ but will also be informed by ideology, values, public opinion and lobbying [7]. Policy-network theory argues that the political relevance of evidence, especially its alignment to prevalent ideological imperatives and political priorities, is fundamental to its reception and potential uptake by policy-makers [8]. Therefore, the practice of public health may benefit greatly from explicit acknowledgement of the political nature of health and the development of the political science of health [9]. Based on these clear theoretical, conceptual and structural links between politics and population health, it is important to synthesise the evidence linking these disciplinary areas. In 2011, Muntaner et al. [10] published a systematic review – the first of its kind – synthesising 73 internationally comparative studies linking four key political themes – the welfare state, political tradition, democracy and globalisation – with population health outcomes.

### The Barnish et al. (2018) review

A substantially updated version of this review was published in 2018 by a different research team and found a total of 176 eligible studies [11]. It considered all studies included by Muntaner et al. [10], which had a search cut-off date of April 2010. Barnish et al. [11] updated the body of literature by searching ten scholarly databases from 2010 until April 2017 and conducting supplementary searches on Google Scholar and in relevant bibliographies (up to November 2017). Following an independent dual review by MSB and RVHN-H, studies were included if they were peer-reviewed English-language scholarly papers or book chapters using an ecological or individual quantitative empirical design in human populations from at least two countries to link the welfare state, political tradition, democracy or globalisation to any population health outcome excluding healthcare spending. The reason for the exclusion of healthcare spending as an outcome is due to circularity introduced by healthcare spending being an important component of the welfare state. Political exposures are political conditions to which a country is exposed at a given time. In order to update the Muntaner et al. [10] review, the Barnish et al. [11] review was restricted to the four types of political exposures that were considered in the Muntaner et al. [10] review: welfare state, political tradition, democracy and globalisation. These are necessarily a subset of all potential political exposures. The definitions of these political exposures are provided in Table 1, and were identical to the exposure conceptualisations used in Muntaner et al. [10].

**Table 1:** Definition of political exposure variables

Exposure variable	Definition
Welfare state	**“**if the analysis included welfare regimes or welfare state indicators (e.g. universal health coverage), but not measures of political ideology (e.g. along the left-right dimension)**”**
Political tradition	**“**if the study included variables referring to the left-right political dimension (e.g. social democratic / egalitarian/ left vs. liberal / conservative / right political parties in government)**”**
Democracy	**“**if the hypotheses tested involved democratic institutions or political rights**”**
Globalisation	**“**if the article examined how high, middle, and/or low income countries are integrated through global networks of trade, foreign investment, and multinational corporations**”**

Adapted from Barnish et al. (2018), Table 1. Copyright: Max Barnish, Michelle Tørnes and Becky Nelson-Horne – used by permission

Compared to the Muntaner et al. [10] review, the updated review by Barnish et al. [11] provided strengthened evidence that a more generous welfare state, left-of-centre political tradition and increased democracy were all associated overall with superior population health. The evidence for globalisation was on balance in favour of showing that increased globalisation predicted poorer population health. However, the evidence was not consistent with 25% of studies showing the opposite effect and another 25% being inconclusive. The Barnish et al. [11] review did not focus greatly on more specific relationships between the political exposures and particular domains of population health outcomes. Furthermore, it did not investigate the mechanisms by which the effect of political exposures on population health outcomes may operate, as is typical for systematic reviews.

### Relevant theoretical perspectives

Realist synthesis [12] is an approach to evidence synthesis that permits the analysis to proceed beyond description and offer an explanatory insight into how the effects identified in the literature may operate. These theoretical insights are framed in terms of programme theories, which are composed of combinations of context, mechanism and outcome. The theoretical lens of realist synthesis is based on the epistemological foundations of critical realism [13]. A realist synthesis approach allows context specific and temporally sensitive middle range theory to be constructed, enabling the understanding of a wide range of intermingling relations between contexts and mechanisms that leads to favourable or unfavourable population health outcomes.

There are a number of macro-theoretical perspectives that may inform considerations around the relationship between politics and population health. Neoliberalism represents a twentieth century resurgence of nineteenth century ideas favouring laissez-faire economic liberalism and free market capitalism [14] accompanied by austerity and minimal government intervention to secure social, economic and public health goals. A dominant force in western politics since the 1980s, this theoretical perspective has featured widely in scholarly discourse linking politics and population health. For example, an analysis of the health consequences of Thatcherism in the United Kingdom was conducted by Scott-Samuel et al. [15], a conceptualisation of neoliberalism – along with economic globalisation – as a key driver of health inequity [16], and a book on ‘neoliberal epidemics’ has been published [17]. A qualitative case study by Garnham [18] offers further insight into how the effects of neoliberalism may operate, although this study is limited to post-industrial west central Scotland, and context may be important in terms of how political exposures have their impact.

Another important macro-theoretical perspective to consider is dependency theory – the notion that through international trade, resources flow from a ‘periphery’ of less affluent and less developed countries to a core of affluent states. This results in a flow of economic resource from less affluent to more affluent countries, with more affluent countries being enriched and less affluent ones impoverished by the roles through which they fit into the world’s economic system. Though seen as limited as an overall theory, dependency theory retains relevance as a conceptual orientation to understand the global division of wealth [19]. Nevertheless, the applicability of dependency theory to developed countries may be limited by the shift in many developed countries from an industrial economy to an information, service and hospitality-intensive economy.

Socialist and social democratic theories are also relevant to this topic. Although often used more or less interchangeably in everyday discourse, the common element across the range of divergent definitions of and theoretical perspectives on socialism is social ownership and control of the economy [20]. By contrast, social democracy may have socialism as a long-term goal [21], but embraces a Keynesian mixed economy whereby substantial state intervention in the form of income redistribution, regulation and a welfare state operates within a largely capitalist market economy [22]. Socialist and social democratic perspectives typically have their origins in revolutionary movements of the mid-to-late eighteenth century that arose in response to an awareness of social problems arising from the development of more advanced capitalism [23]. Within the socialist theoretical sphere, for example, a Marxist theoretical lens has been used to critique the role of political power and economic dominance in capitalist society and how health policy interventions reflect different groups’ political and economic goals, how state intervention protects capitalist interests and the private sector, and how medical ideology helps maintain class structure [24]. More recently, the works of Marx and Engels have been used to draw theoretical connections between capitalism and poor health through a critique of the healthcare industry [25], while Horton [26] indeed claims that medicine and Marxism have ‘entangled, intimate, and respectable histories’. Socialist and social democratic theoretical perspectives can inform analysis not solely of health care aspects, but also the state provision of welfare, the extent to which policies are redistributive, and how class interests can come to bear upon policies that influence societal organisation and social provision relevant to population health.

### Aim of the current work

The aim of this work was to provide a realist re-analysis of a large systematically identified body of studies on the relationships between political exposures and child and maternal health outcomes – drawn from the Barnish et al. [11] review, in order to evaluate which theories are best supported by the evidence, since the pathways through which these influences may operate have not been systematically appraised. The use of a dataset from a previously published systematic review [11] is an important validation stage in ensuring a robust body of evidence to serve as a source for realist re-analysis. Child and maternal health were selected as the outcome domains for this realist re-analysis as these outcomes directly reflect a country’s health system’s performance [27]. The World Health Organization noted that 810 women die every day from preventable causes related to pregnancy and childbirth, while the Millennium Development Goal 5 calling for a 75% reduction in maternal mortality and universal access to reproductive health by 2015 was not met, despite substantial improvements [28]. Child and maternal health issues are important in developed countries as well as in low- and middle-income countries (LMECs). For example, the American Public Health Association notes that ‘far too many women, infants and children worldwide still have little or no access to essential, quality health services and education, clean air and water, and adequate sanitation and nutrition’ and that sociological factors play an important role in determining child health outcomes, and that addressing this is important for health equity [29]. Therefore, child and maternal health are important components of population health, and can be seen as important concepts for human rights, and worthy of being the focus of study in this realist re-analysis.

This analysis will generate micro-theoretical initial programme theories by re-reading and undertaking realist engagement with the systematic review publication by Barnish et al. [11] – across all outcomes as presented in the systematic review. These initial programme theories will then be adjudicated in the context of the evidence base for child and maternal health. Finally, the final programme theories that emerge as most strongly supported by the evidence base will be comparatively analysed and situated in the context of the macro-theoretical perspectives.

## Methods

### Identification of evidence

The database of potentially eligible studies for this realist review was drawn from a systematic review on the political determinants of health [11]. Ten scholarly databases were searched: MEDLINE, AMED, EMBASE, PsycINFO, CINAHL, Philosopher’s Index, Science Citation Index Expanded, Social Sciences Citation Index, Emerging Sources Citation Index and Sociological Abstracts. Search strings were developed for each database from the conceptual search strategy shown in Table 2. These used expanded, ‘exploded’ or equivalent index terms as relevant to the database. This means that each search term also automatically searched a range of narrower search terms beneath it in the search tree. For example, on Medline, exp. Health/ also retrieves Child Health/, Women’s Health/ and Reproductive Health/. Supplementary searches were conducted on Google Scholar and relevant bibliographies, with a final search date of November 2017.

**Table 2:** Conceptual search strategy

((democracy OR autocracy OR welfare regime OR welfare state OR welfare capitalism OR politics OR political tradition OR internationality OR globalization) AND (health OR health services OR population health OR public health OR health economics OR health expenditure))	

Reproduced from Barnish et al. (2018), Box 1. Copyright: Max Barnish, Michelle Tørnes and Becky Nelson-Horne – used by permission

Independent dual review (MSB and RVHN-H) was used for all review processes. In the systematic review [11], the following study selection criteria were applied:

- Peer-reviewed journal article in a scientific journal or a scholarly book or chapter
- Study human populations either at the individual or ecological level
- Present at least one measure of a political exposure, conceptualised in terms of the welfare state, political tradition, democracy or globalisation. These political features were defined exactly following Muntaner et al. [10], and listed in Table 1.
- Present at least one measure of a population health outcome. Healthcare spending alone was not considered an eligible outcome
- Use any quantitative empirical design to link the exposure to the outcome
- Present a comparison involving at least two sovereign countries

The set of studies that were included in the systematic review by Barnish et al. [11] were included in this realist review if they contained child and/or maternal health outcomes, due to their correlation with health system performance. This further assessment was conducted by MSB and discussed with GJMT to reach consensus. Data extraction for the systematic review was led by MSB with a proportionate 20% check by RVNH. Where additional data extraction was needed to explore relevant context, mechanism and outcome configurations in the theory adjudication process, this was conducted initially by MSB and checked by GJMT.

### Realist re-analysis

The scope of the realist re-analysis was child and maternal health. In planning a realist synthesis of this nature, it is important to define a scope that is both realistic and meaningful. To this end, it was not possible to include the entirety of the scope of population health, as seen in prior systematic reviews [10, 11] that did not employ realist methods. It was therefore important to select a specific area of population health to form the remit for this realist synthesis. Child and maternal health were selected as the outcome domains for this realist re-analysis as these outcomes directly reflect a country’s health system’s performance [27]. Furthermore, child and maternal health have been identified as key priorities at a policy level. For example, the World Health Organization (WHO) identifies maternal health as “one of WHO’s key priorities” [30] and state that “protecting and improving the health of children is of fundamental importance” [31], while “reducing child mortality” [32] and “improving maternal health” [33] featured among the United Nations’ Millennium Development Goals. Realist analysis followed a standard a priori protocol as described in this section. It was not published. Therefore, we provide detailed description here in the manuscript as to how the realist re-analysis process operated.

The first stage of the realist re-analysis process was to generate micro-theoretical initial programme theories for use in the theory adjudication process. Realist initial programme theory development is typically iterative and informed by a defined set of sources of knowledge, which can include textual sources, discussions, broader epistemological and theoretical perspectives applicable to the broad field of study, and the authors’ subject knowledge. At the very beginning of this iterative process, lead author MSB re-read the systematic review paper and associated appendices [11] three times – once at an overview level, once in detail in a descriptive manner, and once in detail in a theoretically engaged manner. Standardised themes were not imposed upon these initial thoughts in order to avoid inappropriately constraining the initial programme theories that could emerge. Then, through realist engagement [12] with the results of this reading, potential initial programme theories started to emerge. The systematic review covers a broader range of outcomes – all population health outcomes except healthcare expenditure – than this realist review, so initial engagement was not restricted to child and maternal health outcomes. These initial theoretical ideas were then discussed with GJMT as a peer validation process and iterated accordingly. It was at this stage that the authors sought – as per the pre-specified a priori protocol – to focus the emergent theoretical concepts on child and maternal health. The next stage of iteration involved MSB critically engaging with existing knowledge about child and maternal health and also with broader epistemological and theoretical work around the political determinants of health, relevant sources for which are outlined in the introduction under ‘Relevant theoretical perspectives’. Finally, the iterated ideas were again discussed with GJMT, and refined a final time to generate the initial programme theories which were formalised and are presented in this manuscript. This formed the initial programme theories on which theory adjudication was performed. Each programme theory was conceptualised in terms of context, mechanism and outcome configuration. Once initial programme theories were generated, they were then thematically analysed according to standardised themes to align them to one of the four political themes being investigated in this realist synthesis: welfare state, political tradition, democracy or globalisation.

These initial programme theories were then adjudicated in the context of the evidence base for child and maternal health identified for this realist review, which comprised all studies from the Barnish et al. [11] systematic review for which child and/or maternal health outcomes were available. Theory adjudication [34] was performed separately for each programme theory, and was conducted by MSB and verified and refined through iterative rounds of discussion with GJMT. When conducting theory adjudication, initially all studies relevant to the appropriate political exposure were considered in order to assess consistency of results. Once it was established that the results were consistently in the direction that would support the initial programme theory, further and more detailed adjudication was conducted on the set of studies for which the results were in line with this hypothesis. This enabled the precise context, mechanism and outcome configuration to be tested, once the general suitability of the proposition had been established. For example, once it was established that studies consistently supported a beneficial effect of the welfare state on child and/or maternal health outcomes, further detailed adjudication would take place only on those studies that showed a beneficial effect of the welfare state, in order to assess how this effect operated. Theory adjudication was not stratified by developed countries versus LMECs. This decision was taken because the authors considered that the disadvantages of imposing such a stratification outweighed potential advantages. A considerable number of studies include both developed countries and LMECs in a single analysis, so these studies would have to be excluded from a stratified realist synthesis, imposing substantial selection bias. This was known to be the case from the systematic review so could be built into the pre-specified protocol. Such a stratification would also preclude a unified assessment of the evidence base, would preclude assessment of which type of country context is most pertinent to a given theoretical configuration, and would represent a substantial deviation from the systematic review upon which this realist analysis was based. For these reasons, stratification by developed countries and LMECs was not used. Theory adjudication was performed following theory presentation in order to assess which theories, following revision, were best supported by the evidence base.

The theory adjudication process involved several stages. The overall categorical result profile (% studies assessed as positive for each exposure theme as per the assessment method in the systematic review, i.e. across all systematic review outcomes) was compared between the set of studies included in the realist review and the complete set of studies in the systematic review. This provides an assessment of consistency of findings, which is important to consider in the adjudication of which theories are best supported by the evidence. The studies included in the realist review were assessed to determine the extent of coherence between the body of evidence and the outcome of the proposed context, mechanism and outcome configuration – since the categorical result in the systematic review was assessed across all outcomes, not exclusively those related to child or maternal health. If the proposed context, mechanism and outcome configuration appeared still viable based on outcome, the viability of the proposed content and mechanism elements were assessed. It was at the theory adjudication stage that detailed critical engagement with specific individual eligible studies identified by the systematic review took place.

Initial programme theories were revised as required to form the final programme theories. For those initial programme theories that were not supported for child and maternal health following theory adjudication, they were assessed in the totality of the set of studies for the systematic review, in order to determine how well they were supported for population health outcomes more broadly. This is an important step in order to contextualise the findings and assess their generalisability. The final programme theories that emerged as most strongly supported by the child and maternal health evidence base were then comparatively analysed and situated in the context of the macro-theoretical perspectives, as outlined in the introduction under ‘Relevant theoretical perspectives’. At all stages in the theory adjudication process, the initial analysis was conducted by MSB, and this was subjected to peer validation and refinement through discussion with GJMT. This iterative approach based on critical reflection and discussion is an important component of realist theory adjudication. There is not a standard coding software for the type of analysis undertaken. Therefore, analysis was conducted manually.

## Results

### Identification of evidence

The systematic review by Barnish et al. [11] identified a total of 35,333 unique records, of which 255 proceeded to full-text review, and 176 to inclusion. Following further assessment against the additional criterion for the realist review – that the studies included at least one child or maternal health outcome – a total of 67 studies were included in the realist review, while the remaining 109 were excluded (except for the purposes of comparison with the systematic review) due to ineligible outcomes, i.e. they did not contain results related to child and/or maternal health outcomes.

### Study profile

Four studies (6%) involved measurement at the individual level [35–38], while the remaining 63 were ecological. The collective period of data collection across studies was 1950–2014. Duration of data collection ranged across studies from 1 year to 50 years [39, 40] with a median of 15 years. Start or end year of data collection were not available for five studies (7%). Two studies were conducted specifically in the context of former Communist countries [41, 42], while one further study was conducted in the context of countries more generally transitioning to democracy [43]. Eighteen studies (27%) were conducted in exclusively developed countries, while 21 studies (31%) were conducted in exclusively low- and middle-income countries. Twenty-five studies (37%) assessed the welfare state exposure theme, eight studies (12%) assessed political tradition, twenty-eight studies (42%) assessed democracy, and 13 studies (19%) assessed globalisation. Unlike in Muntaner et al. [10], studies were allowed to contribute to more than one exposure theme. A summary of the study profile is provided as Supplementary information 1.

### Initial programme theories

Six initial programme theories were posited for adjudication. These are presented in Table 3.

**Table 3:** Initial and final programme theories

Theory #	Initial programme theory	Final programme theory
1	A more generous welfare state has a beneficial effect on child and maternal health outcomes including infant and maternal mortality (Outcomes) especially in LMECs (Context) by improving the social conditions especially of those who face deprivation and ensuring they have what they need, including through progressive child- and family-facing social policies (Mechanism).	A more generous welfare state has a beneficial effect on child and maternal health outcomes including infant and maternal mortality (Outcomes) especially in developed countries (Context) by improving the social conditions especially of those who face deprivation and through progressive social welfare policies (Mechanism).
2	A left-of-centre political tradition has a beneficial effect on child and maternal health outcomes including infant and maternal mortality (Outcomes) especially in LMECs (Context) by generating a greater focus on welfare state measures, especially progressive child- and family-facing policies, to improve the social conditions especially of those who face deprivation in order to ensure they have what they need (Mechanism).	A left-of-centre political tradition has a beneficial effect on child mortality and low birth weight (Outcomes) especially in developed countries (Context) by generating a greater focus on welfare state measures, especially progressive child- and family-facing policies, to improve the social conditions especially of those who face deprivation in order to ensure they have what they need (Mechanism).
3	Greater democracy has a beneficial effect on child and maternal health outcomes including infant and maternal mortality (Outcomes) especially in LMECs (Context) by promoting empowerment, acting as a safeguard against despotism and through increased accountability facilitating provision of requisite systems and services including progressive child- and family-facing social policies (Mechanism).	Not supported.
4	The introduction of capitalist democracy into a communist autocracy (Context) has a negative short-term effect on child and maternal health outcomes including infant and maternal mortality (Outcomes) by the introduction of marketization and erosion of state networks that support health and minimise health inequalities, such as a roll-back of state-supported progressive child- and family-facing social policies (Mechanism).	Not supported.
5	Increased globalisation has a beneficial effect on child and maternal health outcomes including infant and maternal mortality (Outcomes) especially in LMECs (Context) by increasing prosperity resulting from increased trade with more advanced economies leading to more money being available for public services including the provision of progressive child- and family-facing social policies (Mechanism).	Not supported.
6	Increased globalisation has a negative effect on child health especially obesity and diseases resulting from obesity (Outcome) especially in LMECs (Context) by encouraging exposure to greater commercial interests such as in the food and drink industry, thereby generating an obesogenic environment and increasing the exposure of children to unhealthy products including food and drink (Mechanism).	Increased globalisation has a negative effect on child and infant mortality and youth smoking rates (Outcome) in LMECs (Context) by increased international trade dependency and greater influence of multinational corporations and neoliberally oriented international trade organisations (Mechanism).

*LMECs* Low- and middle-income countries

### Theory adjudication

*Theory 1 – the beneficial effect of the welfare state on child and maternal health especially in LMECs through progressive social policies (*Table 3*).*

There was a total of 25 studies assessing the relationship between the welfare state and child and/or maternal health outcomes. Twenty-two studies (88%) were assessed as positive, i.e. demonstrating evidence that a more generous welfare state led to superior health outcomes. This proportion was comparable to the wider systematic review by Barnish et al. [11] where the corresponding figure was 79 out of 102 (77%). Welfare state provision may be conceptualised using a range of classification schemes. One influential work is The Three Worlds of Welfare Capitalism [44]. This broadly categorises welfare states into neoliberal regimes, social democratic regimes and residual regimes, the latter being characterised by family-first support and a minimalist welfare regime. Of these 22 studies, 21 [27, 36, 38–40, 45–60] remained classified as positive when only child and maternal health outcomes were considered. In the study by Klomp and de Haan [61], the outcomes were based on 16 national health indicators that cover child and maternal health aspects, although the analytic model is written up in a way that does not permit the separable effect of the welfare state variables on the child and maternal health outcomes to be distinguished.

Out of the initial 25 studies, 21 studies reported a positive association between more generous welfare state provision and more positive child and maternal health outcomes, and were subjected to more detailed theory adjudication. Among these 21 studies, 12 assessed infant mortality outcomes [39, 40, 45, 46, 48–50, 52–54, 56, 57], five assessed under-five mortality [40, 45, 49, 51, 60], four studies assessed each of maternal mortality [27, 45, 47, 56], low birth weight [40, 47–49] and child mortality [27, 36, 53, 55, 60], three studies assessed child poverty [36, 53, 56] and one study assessed each of child wellbeing [58], fertility [59] and neonatal mortality [38]. Therefore, both child and maternal health were assessed. Among these 21 studies, there was greater focus on developed countries than LMECs. There were five studies that focused exclusively on LMECs [38, 45, 51, 53, 57], while six studies considered both developed countries and LMECs [27, 40, 54–56, 60], and the remaining ten studies included only developed countries. Studies differed with regard to how they assessed the welfare state – either in terms of welfare state typology classification or welfare state financial expenditure – but the differential effect on outcomes was small. Compared to the systematic review, a higher proportion of studies included in this realist review used an expenditure-based approach. Studies on LMECs all considered welfare state in terms of health expenditure and/or its impact on healthcare coverage. Studies on developed countries, or those that included both developed countries and LMECs varied in terms of the use of regime or expenditure-based methods. Studies by Wu and Chiang [60] on developed countries and LMECs and by Chung and Muntaner [49] considered social services expenditures rather than solely health expenditures. Studies using regime classifications frequently cite Esping-Andersen [44] as an important influence, but exact classification schemes do differ. For example, Karim et al. [54] assessing both developed countries and LMECs used a six-way classification scheme: Scandinavian, Anglo-Saxon, Bismarckian, Southern, Eastern European and East Asian” which contrasts for example with a simpler three-way system: Liberal, Conservative and Social Democratic used by Bambra [46] or four-way system: Social Democratic, Christian Democratic, Liberal and Wage Earner used by Chung and Muntaner [48], both in developed countries.

Across studies, there was consistent evidence to support the proposed mechanism being more generous welfare policies to improve the social conditions of the deprived, although it is noted that studies did not consistently provide sufficient granularity of information on welfare state exposures to determine exactly which policies were effective. It was clear across studies that greater expenditure on welfare state measures (in studies assessing welfare state financial expenditure) and approaches to welfare state provision corresponding to the social-democratic classification in Esping-Anderson [44] predicted the best outcomes. A revised version of theory 1 is presented (Table 3).

*Theory 2 – the beneficial effect of left-of-centre political tradition on child and maternal health outcomes especially in LMECS through a greater focus on progressive policies (*Table 3*).*

There was a total of eight studies assessing the relationship between political tradition and child and/or maternal health outcomes. All eight studies (100%) were assessed as positive, i.e. demonstrating that left-of-centre political tradition led to superior health outcomes. This proportion was higher than in the wider systematic review by Barnish et al. [11] where the corresponding figure was 15 out of 17 (88%). Of these eight studies, seven [49, 62–67] remained classified as positive when only child and maternal health outcomes were considered. In the study by London and Williams [68], the child and maternal health aspects of the composite index of 41 indicators of domestic well-being were not presented separately.

Among these seven studies, six assessed infant mortality [49, 62, 63, 65–67], two assessed each of child mortality [62, 64] and low birth weight [49, 64] and one assessed under five mortality [49]. These studies did not assess maternal health. Contrary to expectation, the evidence base was not concentrated largely in LMECs, but instead rather largely in developed countries. Lena and London [62] assessed periphery and non-core nations, Moon and Dixon [63] assessed a range of countries without restriction by level of economic development, while the remaining four studies were conducted exclusively in OECD or otherwise wealthy countries. As anticipated, the evidence base focused on different aspects of child mortality, although two studies also assessed low birth weight – another aspect of debility.

Regarding mechanisms, the beneficial effect of left-of-centre political tradition operating via an increased focus on the welfare state was supported by all six studies. For example, Chung and Muntaner [49] concluded that “strong political will that advocates for more egalitarian welfare policies, including public medical services, is important in maintaining and improving the nation’s health”. Lena and London [62] demonstrate that political systems exert an influence on health and well-being independent of national and international economic factors. This adds further weight to the argument that it is political will that makes the difference rather than solely affluence – since it determines how a country’s resources are deployed, and whether or not it is in ways that benefit population health. Moon and Dixon [63] explore a slightly different aspect of the same conceptual phenomenon and find that in left-wing countries state strength promotes welfare performance, while in right-wing countries it inhibits the provision of basic needs, again emphasising the importance of political will. Moreover, political will may also be related to political capacity [69, 70], which is the ability to exert reflexive policy learning when navigating a complex policy environment that is laden with multiple interests from multiple stakeholders. Muntaner et al. [64] also emphasise the role of ideological outlook in promoting a strong welfare state. Work by Navarro and Shi [67] and Navarro et al. [65, 66] furthermore emphasises the role of levels of public expenditures and health care benefits coverage and degree of redistributional focus. More specifically, they emphasise additionally public support of services to children as an important predictor of health status, and an important mechanism by which the positive effect of left-of-centre political tradition on child health outcomes may operate. None of the seven studies assessed maternal health. A revised version of theory 2 is presented (Table 3).

*Theory 3 – beneficial effect of democracy on child and maternal health outcomes especially in LMECs by promoting empowerment and provision (*Table 3*).*

There was a total of 28 studies assessing the relationship between democracy and child and/or maternal health outcomes. Twenty studies (71%) were assessed as positive, i.e. demonstrating that greater democracy led to superior health outcomes. This proportion was comparable with the wider systematic review by Barnish et al. [11] where the corresponding figure was 34 out of 44 (77%). Of these 20 studies, 16 [37–38, 58–59, 67–78) remained positive when only child and maternal health outcomes were considered, while one study [71] became inconclusive and three studies [68, 72, 73] did not present the analysis in a way that enabled the impact on child and maternal health to be assessed separately. Studies tended to include a large range of countries and this range often included the USA: a potential caveat regarding the beneficial effect of democracy relates to the USA where there is a high level of democracy, but a highly neoliberal health system in which a high proportion of the population lack insurance coverage. Among these 16 studies, 14 assessed infant mortality [42, 43, 62, 63, 74–83], three assessed each of maternal mortality [42, 75, 79] and child mortality [62, 84, 85], two assessed fertility [79, 84] and the same single study [79] assessed each of receipt of prenatal care, skilled birth attendance, under five underweight, anaemia during pregnancy, haemoglobin levels during reproductive age, tetanus immunization among pregnant women and birth rate among women aged 15–19. The evidence is clear across studies that increased democracy is beneficial for both child and maternal health. However, studies relevant to this theme tended to provide too little information to allow a specific theory to be supported with regard to the mechanism by which democracy achieves a benefit for child and maternal health.

*Theory 4 – negative short-term effect of the introduction of capitalist democracy in communist autocracies on child and maternal health outcomes through the erosion of state networks (*Table 3*).*

There was a total of 28 studies assessing the relationship between democracy and child and/or maternal health outcomes. One study (4%) was assessed as negative, i.e. demonstrating evidence that greater democracy led to inferior health outcomes [41]. This proportion was comparable to the wider systematic review by Barnish et al. [11] where the corresponding figure was two out of 45 (5%).

However, this study [41] was no longer assessed as negative when only child and maternal health outcomes were considered. Instead, infant mortality rates were shown to follow the global trend, i.e. fell as income rose, with increased income resulting from greater democratisation in the context of the transition from Communism in Central and Eastern Europe. Therefore, theory four was not supported given there was no evidence that increased democracy resulted in inferior child and/or maternal health outcomes.

By means of comparison, in the entire set of studies and outcomes from the systematic review, there is evidence to suggest that the introduction of democracy may have a negative effect on health by disrupting existing systems that underpin health at least in the short term. Gauri and Khaleghian [86] found that increased democracy predicted reduced diphtheria, tetanus, pertussis and measles vaccine coverage in 2018 LMECs with the erosion of state networks of compliance likely to be a key factor. Meanwhile, Adeyi et al. [41] found that increased democracy in the Central and Eastern European transition context exerted a negative effect on the probability of dying between 15 and 65 years. The authors explain that reduced life expectancy at birth results from rising income level being associated with an increased probability of death between the ages of 15 and 65, stating that in this context “the wealthier the society, the less healthy is its population, particularly for its males” [41]. Systems factors were likely to play a key role, and it is also important to note the temporal effect.

*Theory 5 – beneficial effect of globalisation on child and maternal health outcomes especially in LMECs through increased prosperity benefitting public services (*Table 3*).*

There was a total of 13 studies that assessed the relationship between globalisation and child and/or maternal health outcomes. Five studies (38%) were assessed as positive, i.e. demonstrating evidence that greater globalisation led to superior health outcomes. This proportion was higher than in the wider systematic review by Barnish et al. [11] where the corresponding figure was seven out of 28 (25%). Of these five studies, all [83–87] remained classified as positive when only child and maternal health outcomes were considered.

Among these five studies, all assessed infant mortality and one study each assessed child mortality [86] and under five mortality [87]. Therefore, all outcomes related beneficially to globalisation were related to different aspects of child mortality, and none assessed maternal health. All five studies assessed a wide range of countries with a range of different world system roles and were not restricted to LMECs. The evidence does not appear to support the suggestion that the positive effects of globalisation are largely limited to LMECs. Gerring and Thacker [88] explicitly consider the direct effects of globalisation rather than those indirectly routed via economic growth. This study found that openness to imports and long-term membership in neoliberally oriented international trade bodies are associated with lower rates of infant mortality at a global level when the analysis controls for general economic performance, as measured by GDP per capita. However, the authors acknowledge an inability to determine causal mechanisms and any theoretical claims in their paper are recognised as largely speculative. Whereas Owen and Wu [89] and Moore et al. [90] focus on economic aspects of globalisation, Mukherjee and Krieckhaus [91] and Martens et al. [87] exemplify important social and political aspects of globalisation playing a role in the benefit of globalisation for population health.

Across studies, there appears little consensus on how the potential positive impact of globalisation on population health may operate and no specific theory can be supported at this current time. This situation is comparable across the full range of systematic review outcomes. Additionally, authors also acknowledge that neoliberal policies create winners and losers and increase health inequalities, so care needs to be taken in the interpretation of evidence regarding the potential benefits of globalisation, especially when no single theory gains much empirical support.

*Theory 6 – negative effect of globalisation on child health especially in LMECs by generating a commercially-driven obesogenic environment (*Table 3*).*

There was a total of 13 studies assessing the relationship between globalisation and child and/or maternal health outcomes. Five studies (38%) were assessed as negative, i.e. demonstrating evidence that greater globalisation led to inferior health outcomes. This proportion was lower than in the wider systematic review by Barnish et al. [11] where the corresponding figure was 14 out of 28. Of these five studies, all [82, 92–95] remained classified as negative when only child and maternal health outcomes were considered, although it should be noted that in Fan & Le’au [92], the effect was only found for neonatal rather than infant mortality.

Among these five studies, two each assessed infant mortality [82, 94] and neonatal mortality [92, 95] and one each assessed child mortality [92] and youth smoking [93]. None assessed maternal health. Whereas obesity was a common outcome among studies in the wider systematic review, in line with the frequent conceptualisation of the negative impact of globalization and trade liberalisation in terms of an obesogenic environment, obesity was not a measured outcome in any of the studies specifically in the child and maternal health context. While Fan & Le’au [92] found support for a negative impact of increased globalisation on neonatal mortality, obesity and overweight were only assessed in an adult population. Therefore, theory 6, focusing on obesity, was not supported by the evidence. However, some of the broader conceptual points behind this theory found support in the evidence. In the context of youth smoking, Maynard [93] provides support for the idea that increasing international trade dependency and membership in neoliberally oriented international trade bodies leads to increased youth smoking by increasing the power of multinational companies in the LMEC context resulting in greater availability and promotion of tobacco and weaker tobacco control policies. All the five studies considered exclusively the LMEC context, which is coherent with a theory proposing that the negative effects of globalisation on health largely operate in an LMEC context. Economic aspects of globalisation were dominant, also being the focus of Shandra et al. [82], Shen and Williamson [94] and Shen and Williamson [95], which linked aspects of increased trade dependency to child and infant mortality. This conceptualisation is referred to in the literature as dependency theory. However, Fan and Le’au [92] show that social and political aspects of globalisation may also play a role. Across studies, a revised version of theory 6 (see Table 3) gains substantial support, with economic factors clearly dominating the evidence base.

### Comparative evaluation

**Fig. 1:**
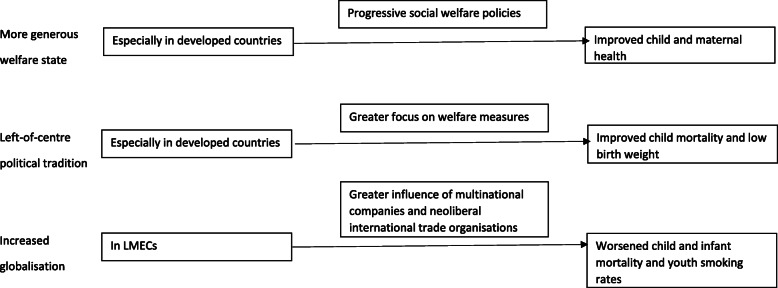
Depiction of final programme theories. LMECs = low- and middle-income countries. Text on the left of the figure refers to the political exposure theme. The left box is the context. The right box is the outcomes. The box above the arrows is the mechanism

Three final programme theories were supported in the evidence base for child and maternal health outcomes following realist re-analysis (Fig. 1). These were revised versions of theories one, two and six (Table 3). Final programme theory one – relating to the welfare state – was the theory that attracted the greatest support, noting that theories assess different aspects of political exposures and are not mutually exclusive. Theory two – relating to political tradition – attracted the next greatest support. The evidence base for theory two was small but consistent. Far fewer studies assessed political tradition explicitly than the welfare state. The welfare state can be seen as a marker for political tradition in so far as political tradition, preferences, ideology and associated political will drive the extent to which a generous welfare state is offered. Theory six – relating to the negative effect of (primarily economic) globalisation – was supported, but the evidence base was relatively small. Theory six can be related to macro-theoretical perspectives relating to dependency theory, and work in this area is typically clearly situated in this theoretical perspective. With regard to political tradition and the welfare state, the final programme theories supported align with socialist and social democratic macro-theoretical perspectives that favour state intervention to secure social and health goals rather than neoliberal perspectives that favour economic freedom and minimal state intervention [96]. As per the scope of this realist review as described in the methods section, the three final programme theories supported all relate to child and maternal health, as opposed to population health more generally. In theory one, relating to the welfare state, the focus is on infant and maternal mortality, which encompasses both child and maternal health. In theory two, relating to political tradition, the focus is on child mortality and low birth weight, thereby taking a child health perspective. In theory six, relating to globalisation, a child health perspective is again taken, with a broader range of key outcomes, including child and infant mortality, as well as youth smoking rates (age 11–17).

## Discussion

This article presents a realist re-analysis focusing on child and maternal health outcomes and identifies that several theories relating to different aspects of the relationship between political exposures and child and maternal health outcomes can be supported. In comparative evaluation, evidence for final programme theory one relating to the welfare state was the strongest. This could be related back to socialist and social democratic macro-theoretical perspectives that emphasise ‘socialised medicine’ and a greater focus on state-led social provision and redistributive efforts. The effect of the welfare state may be stronger in developed countries, which may reflect a context effect related to the ability to deploy resources for the benefit of health. This realist analysis provides insight into how the beneficial effects of a more advanced welfare state and left-of centre political tradition, and the negative effects of increased globalisation on child and maternal health may operate. Furthermore, temporality may account for different mechanisms and outcomes that we observe in the context of democracy. It should be noted that it cannot be guaranteed that the same context mechanism and outcome configurations are applicable to other population health outcomes. This is because realist programme theories are highly sensitive to the relationship between particular combinations of contexts, mechanisms and outcomes.

The welfare state is an important concept in social scientific discourse relating to population health and a more advanced welfare state has been identified as an important determinant of better population health across a wide range of population health outcomes by evidence syntheses by Barnish et al. [11], Muntaner et al. [10] and Berqvist et al. [97]. Studies in the current work differed in terms of how welfare state generosity was assessed. Studies largely subdivided into two key approaches – those categorising welfare state provision according to a classification typology such as Esping-Anderson [44] and those directly assessing financial expenditure. It remains unknown whether one methodological approach is superior to the other. Berqvist et al. [97] found that welfare state financial expenditure was a better predictor of population health outcomes than welfare state classification typology, while Barnish et al. [11] found the effect to be in this direction but small in magnitude. Studies assessing welfare state financial expenditure often assessed this expenditure in terms of Gross Domestic Product (GDP), assessing the degree of relative focus on welfare state expenditure. Nevertheless, it is clear that both aspects of welfare state provision are predictors of population health, so the context, mechanism and outcome configuration for welfare state in the current analysis did not need to be stratified according to how the welfare state exposure had been measured. Unlike a realist synthesis by O’Campo et al. [98], psychological mechanisms for the operation of the welfare state were not proposed through engagement with the literature nor identified as supported through in-depth realist evaluation of the included dataset. These differences are likely to relate to differences in the dataset. O’Campo et al. [98] considered only adult health, while the current work considers child and maternal health. Moreover, O’Campo et al. [98] considered only OECD countries, while the current work considers a full spectrum provided that the study includes data from at least two countries. Furthermore, O’Campo et al. [98] focused on unemployment insurance schemes rather than the welfare state more broadly. These differences show that realist evaluation is sensitive to differences in context as well as eligible exposures and outcomes. Two further final programme theories were also supported – relating to political tradition and the negative effects of economic globalisation – although the evidence base was not as strong as for the welfare state. It was notable that far fewer studies, both in this realist re-analysis and in the systematic review by Barnish et al. [11], assessed political tradition explicitly than assessed the welfare state, even though the latter can be considered a marker of the former.

The definition of population health has evolved over recent decades and continues to evolve given the recent emphasis on social determinants of health [99] and the concept of ‘health in all policies’ [100], which have shaped how population health is conceptualised. Whereas a more traditional conceptualisation of population health may be along the lines of “the health outcomes of a group of individuals, including the distribution of such outcomes within the group” [101], latest conceptualisations of population health may encompass aspects as diverse as education and town planning, which are operationally quite separate from health services [102].

### Strengths and limitations

The presented work has several key strengths. It uses realist synthesis, based on the theoretical lens of critical realism, to offer an explanatory perspective to inform considerations around the relationships between political exposures and population health. This generated micro-theoretical initial programme theories based on a published systematic review by Barnish et al. [11], adjudicated and revised them in line with the evidence base for child and maternal health outcomes among included studies, and situated them in the context of macro-theoretical perspectives from the wider literature. Through using a set of studies from a published systematic review, further assessed to include only those addressing child or maternal health, this realist re-analysis benefits from the strengths of the original systematic review especially with regard to systematic searching and evaluation of studies for inclusion, and the use of independent dual review, in order to minimise the potential of reviewer bias [103]. The search strategy included ten major scholarly databases plus appropriate supplementary searches to maximise coverage. Moreover, the Barnish et al. [11] review is the largest systematic review available on the political determinants of health, so provides a particularly apt source of data for realist re-analysis. The internationally comparative perspective –requiring eligible studies to include data from at least two sovereign countries – taken additionally transcends the particularities of individual countries and improves external validity of the analysis.

However, there are also some limitations that should be taken into consideration. The internationally comparative perspective also introduces complexities in the mapping between political exposures and political parties in both systematic and non-systematic ways [11]. In order to maintain consistency with Muntaner et al. [10], Barnish et al. [11] restricted eligibility to English language sources and peer-reviewed publications, and these restrictions therefore feed through to this realist re-analysis. Furthermore, while a number of key macro-theoretical perspectives were considered, this work was not intended as a compendium of political theory and it was not possible to consider every potential perspective. As a result of using the dataset from a previous systematic review as a quality assurance measure, one trade-off is that the search is not as up to date as would be ideal. A follow-up study in the future could attempt an update from newer studies related to the scopes of the review to bolster the theoretical insights derived from this review. While we considered that overall the disadvantages of stratifying the synthesis by LMECs vs developing countries would outweigh the advantages, it is important to note that a stratified analysis could have offered certain benefits given differences in the social, political, economic and cultural contexts as well as the type and availability of resources between developed countries and developing countries. It was not feasible to undertake a realist synthesis across the full scope of population health from previous systematic reviews that did not employ realist methods. This necessitated a focus on a specific area of population health, for which child and maternal health were chosen, for reasons outlined at the start of the methods section. Exploration of other outcome domains such as mental health, obesity, injury and violence, cardiovascular health, and general health such as life expectancy, would be an area for future work.

There are also some limitations that relate to the available body of evidence rather than the evidence synthesis process. The evidence base is observational, which poses challenges for drawing causal inferences, although a critical realist theoretical lens can help mitigate this. Furthermore, most studies were ecological, which poses an additional limitation regarding the interpretation of results due to the potential of ecological fallacy in extrapolating group-level effects to constituent individuals. As noted by Barnish et al. [11], studies were limited in the thoroughness of their reporting of study design. Fewer studies assessed political tradition explicitly than assessed the welfare state, which may be seen as a marker of political tradition. Outcomes were not assessed consistently across exposure themes. Moreover, maternal health received less attention than child health in the evidence base. Since it used the dataset from Barnish et al. [11], the current realist review could not consider all possible types of political exposures. Instead, it could only consider the welfare state, political tradition, democracy and globalisation. Especially with regard to theories one and two, developed countries were overrepresented in the evidence base, which is a limitation with regard to the generalisability of theories. While fertility as an important component of maternal health was an eligible outcome, the interpretation of fertility results can be context sensitive depending on levels of population growth, which may represent a limitation in the evidence base.

### Future directions

The study and practice of public health stands to benefit from further critically informed works that explicitly acknowledge the political nature of health [9]. Perspectives informed by critical realism – such as realist reviews – may be particularly informative for understanding and advancing the social epidemiology of health [104]. It is important that health research increasingly focuses on real world contexts [4], including the social, cultural and political determinants, since research that ignores these factors may lack relevance and applicability [105]. A critically informed perspective on the social and political science of health offers an opportunity to reflect on the role and remit of public health academia. Muntaner [104] emphasises that social interventions should be undertaken in partnership with affected populations. Schafer [106] reflects on the value of community-academic partnerships in effecting social change, which may be particularly relevant in the context of policy-network theory [8]. Smith and Stewart [107] and Capewell et al. [108] offer reflections on the relationship between academia and practical action. A synergy of these two elements may be important for an applied political science of health to build upon critically informed evidence syntheses such as the one presented in the current work.

Findings highlighted the need to consider how different mechanisms can be activated under different political traditions and welfare regimes in different temporal sequence. These configurations that permutate with time highlight the multi-scalar and non-linear approach in understanding population health. These insights are important for countries in deciding on the viability of future health policies designed to improve population health. In terms of future research, it would be valuable for future work to consider indirect, e.g. second and third order effects, since the current work is limited to consideration of direct effects. Additionally, future research could investigate a broader range of political exposures and pertinent issues, including governance, political culture, whether items have been on the political agenda and whether there has been a policy entrepreneur championing the case, levels of productivity, innovation, and inequity, as discussed for example by Taeihagh [109]. Considering future research directions for primary epidemiological studies, it is important to increase the focus on maternal health outcomes. From a methodological perspective, it could be valuable to explore potential options for using qualitative analysis software, such as NVivo (Melbourne, Australia: QSR International) or Atlas.ti (Berlin, Germany: Atlas.ti) in the context of realist reviews. This is a purpose for which it is not typically currently used, but for which it may offer potential.

## Conclusions

In conclusion, we present a realist re-analysis of a large systematically identified body of evidence on how four key political exposures – the welfare state, democracy, political tradition and globalisation – relate to child and maternal health outcomes. Six initial programme theories were posited. Following realist engagement and re-analysis, which selected the theories that were best aligned to the evidence base and revised them accordingly, three final revised programme theories emerged. Firstly, A more generous welfare state has a beneficial effect on child and maternal health outcomes including infant and maternal mortality (Outcomes) especially in developed countries (Context) by improving the social conditions especially of those who face deprivation and through progressive social welfare policies (Mechanism). Secondly, a left-of-centre political tradition has a beneficial effect on child mortality and low birth weight (Outcomes) especially in developed countries (Context) by generating a greater focus on welfare state measures, especially progressive child- and family-facing policies, to improve the social conditions especially of those who face deprivation in order to ensure they have what they need (Mechanism). Thirdly, increased globalisation has a negative effect on child and infant mortality and youth smoking rates (Outcome) in LMECs (Context) by increased international trade dependency and greater influence of multinational corporations and neoliberally oriented international trade organisations (Mechanism).

## Supplementary Information

**Additional file 1.**

## Data Availability

This realist re-synthesis draws upon data from the Barnish et al. [11] systematic review, which is reported extensively in the publication and its appendices. The results of the realist re-synthesis are presented in the current manuscript. No further data or materials were generated beyond what are presented. Enquiries on the interpretation of the data may be directed to the corresponding author.
