# Supplementary material for: Linking political exposures to child and maternal health outcomes: a realist review
Source: BMC Public Health. 2021 Jan 12;21:127. doi: 10.1186/s12889-021-10176-2 (PMC7802227; doi:10.1186/s12889-021-10176-2)
Supplement: Supplementary file 1 — Additional file 1. [file 12889_2021_10176_MOESM1_ESM.docx]

Supplementary information 1. **Profile of included studies.**

| **Name** | **Year** | **Design** | **Countries** |
| --- | --- | --- | --- |
| Adeyi, 1997 | 1989-1993 | Ecological | 10 former Communist countries |
| Akinci et al., 2014 | 1990-2010 | Ecological | 19 Middle Eastern and North African countries |
| Alvarez-Dardet, 2006 | 2000 | Ecological | 23 former Communist countries |
| Bambra, 2006 | 1980-1998 | Ecological | 18 OECD countries |
| Besley and Kudamatsu, 2006 | 1962-2002 | Ecological | Up to 160 countries transitioning to democracy |
| Bradley et al., 2011 | 2009 | Ecological | 30 OECD countries |
| Bremberg, 2016 | 1990-2012 | Ecological | 28 OECD countries |
| Burroway, 2016 | 1995-2008 | Individual | 52 developing countries |
| Chuang et al., 2013a | 1980-2009 | Ecological | 46 less-developed countries |
| Chuang et al., 2013b | 1980-2009 | Ecological | 46 less-developed countries |
| Chung and Muntaner, 2006 | 1960-1994 | Ecological | 19 OECD countries |
| Chung and Muntaner, 2007 | 1960-1998 | Ecological | 18 wealthy countries |
| Conley and Springer, 2001 | 1960-1992 | Ecological | 19 OECD countries |
| Corsi and Subramanian, 2014 | 1990-2012 | Ecological | 35 sub-Saharan African countries |
| Dietrich and Bernhard, 2015 | 1980s to 2012 | Ecological | 88 countries that were not OECD members in 1984 |
| Elola et al., 1995 | 1990-1991 | Ecological | 17 Western European countries |
| Engster and Stensöta, 2011 | 1995-2005 | Individual | Participants from 20 OECD countries |
| Fan and Faioso Le’au, 2015 | Up to 2014 | Ecological | Independent and American Samoa |
| Fayissa, 2001 | 1993 | Ecological | 34 sub-Saharan African countries |
| Franco, 2004 | 1998 | Ecological | 170 high, middle- and low-income countries |
| Frey and Al-Roumi, 1999 | 1970-1990 | Ecological | 87 developed and less-developed countries |
| Fritzell et al., 2012 | 2000-2005 | Individual | Randomly sampled British, Italian and Swedish mothers |
| Gerring and Thacker, 2008 | 1960-1999 | Ecological | All countries with available data |
| Houweling et al., 2005 | 1999 | Ecological | 43 developing countries in Asia, Africa and Latin America |
| Karim, 2010 | 2003 | Ecological | 30 countries in Europe, North America, Australia and Asia |
| Kick et al., 1990 | 1970-1985 | Ecological | 63 developing countries |
| Klomp and de Haan, 2008 | 2000-2005 | Ecological | 101 low, middle and high income countries |
| Klomp and de Haan, 2009 | 2000-2005 | Ecological | 171 countries with a population greater than 200,000 |
| Kudamatsu, 2012 | Up to 2004 | Ecological | Sub-Saharan African countries |
| Lake and Baum, 2001 | 1970-1992 | Ecological | Up to 110 developed countries |
| Lena and London, 1993 | 1983 | Ecological | Up to 84 peripheral and non-core nations |
| Levine and Rothman, 2006 | Up to 1990 | Ecological | Up to 130 countries |
| Lin et al., 2014 | 1996-2010 | Ecological | 149 countries |
| London and Williams, 1990 | 1965-1970 | Ecological | Up to 110 periphery and semi-periphery nations |
| Lundberg et al., 2008 | 1950-2000 | Ecological | 18 OECD countries |
| Martens et al., 2010 | Up to 2008 | Ecological | Global, subject to data availability |
| Maynard, 2015 | 2000-2010 | Ecological | Up to 85 low- and middle-income countries |
| McKinnon et al., 2016 | 2006-2012 | Individual | Participants from 48 low- and middle-income countries |
| Moon and Dixon, 1985 | 1970-1975 | Ecological | 116 nations |
| Moore et al., 2006 | 2000 | Ecological | 128 countries divided into 6 world-system blocks |
| Mukherjee and Krieckhaus, 2011 | 1970-2007 | Ecological | 132 countries |
| Muldoon et al., 2011 | 2001-2008 | Ecological | 136 United Nations countries |
| Muntaner et al., 2002 | 1989-1992 | Ecological | 16 wealthy countries |
| Navarro and Shi, 2001 | 1960-1996 | Ecological | 18 OECD countries |
| Navarro et al., 2003 | 1950-1998 | Ecological | 17 OECD countries |
| Navarro et al., 2006 | 1972-1996 | Ecological | 17 OECD countries |
| Navia and Zweifel, 2003 | 1990-1997 | Ecological | 188 democratic or dictatorial countries |
| Novignon et al., 2012 | 1995-2010 | Ecological | 44 Sub-Saharan African countries |
| Owen and Wu, 2007 | 1960-1995 | Ecological | 219 countries |
| Pickett and Wilkinson, 2007 | 1998-2006 | Ecological | 23 rich countries |
| Pillai and Gupta, 2006 | 2001 | Ecological | 129 developing countries |
| Pinzón-Flórez et al., 2015 | 2000-2010 | Ecological | 154 countries |
| Ross, 2006 | 1970-2000 | Ecological | 168 countries with a population greater than 200,000 |
| Rovny, 2011 | 1990-1999 | Ecological | 17 OECD countries |
| Rudra and Haggard, 2005 | 1975-1997 | Ecological | 57 less developed countries |
| Safaei, 2006 | 2003 | Ecological | 118 autocratic, incoherent and democratic countries |
| Shandra et al., 2004 | 1980-1997 | Ecological | 59 developing countries |
| Shandra et al., 2010 | 1990-2005 | Ecological | 74 low income countries |
| Shen and Williamson, 1997 | 1960-1991 | Ecological | 86 less developed countries |
| Shen and Williamson, 2001 | 1965-1991 | Ecological | 82 less developed countries |
| Shim, 2015 | 1980-2010 | Ecological | 19 OECD countries |
| Stroup, 2007 | 1980-2000 | Ecological | Up to 105 countries |
| Tsai, 2006 | 1975-1998 | Ecological | 119 developing countries |
| Van der Heuvel et al., 2013 | 1950-2000 | Ecological | Sweden, Netherlands, Canada, USA, Cuba |
| Wejnert, 2008 | 1970-2005 | Ecological | 58 core and peripheral countries |
| Wu and Chiang,2007 | 2002 | Ecological | Taiwan and 21 comparison industrialized countries |
| Zweifel and Navia, 2000 | 1950-1990 | Ecological | 138 democratic or dictatorial countries |

Further study-level details are available in the appendices to the Barnish et al. [11] paper.
